# Action-related eye measures to assess surgical expertise

**DOI:** 10.1093/bjsopen/zrab068

**Published:** 2021-09-03

**Authors:** B Zheng, X Jiang, R Bednarik, M S Atkins

**Affiliations:** Department of Surgery, University of Alberta, Canada; Computing Science, Memorial University of Newfoundland, Newfoundland, Canada; School of Computing, University of Eastern Finland, Joensuu, Finland; Computing Science, Simon Fraser University, British Columbia, Canada

## Abstract

**Background:**

Eye-tracking offers a new list of performance measures for surgeons. Previous studies of eye-tracking have reported that action-related fixation is a good measuring tool for elite task performers. Other measures, including early eye engagement to target and early eye disengagement from the previous subtask, were also reported to distinguish between different expertise levels. These parameters were examined during laparoscopic surgery simulations in the present study, with a goal to identify the most useful measures for distinguishing surgical expertise.

**Methods:**

Surgical operators, including experienced surgeons (expert), residents (intermediate), and university students (novice), were required to perform a laparoscopic task involving reaching, grasping, and loading, while their eye movements and performance videos were recorded. Spatiotemporal features of eye–hand coordination and action-related fixation were calculated and compared among the groups.

**Results:**

The study included five experienced surgeons, seven residents, and 14 novices. Overall, experts performed tasks faster than novices. Examining eye–hand coordination on each subtask, it was found that experts managed to disengage their eyes earlier from the previous subtask, whereas novices disengaged their eyes from previous subtask with a significant delay. Early eye engagement to the current subtask was observed for all operators. There was no difference in action-related fixation between experienced surgeons and novices. Disengage time was strongly associated with the surgical experience score of the operators, better than both early-engage time and action-related fixation.

**Conclusion:**

The spatiotemporal features of surgeons’ eye–hand coordination can be used to assess level of surgical experience.

## Teaser

This study evaluated the most useful action-related eye measures for distinguishing surgeon’s skill levels. Early eye disengagement from previous action was strongly associated with the surgeon’s experience level, which could serve as an optimal behavioural marker for describing surgical expertise.

## Introduction

Surgical expertise is developed through the repeated practice of surgical tasks^1^^,^^2^. For many years, surgical expertise has been measured by peer surgeons using paper-based assessment forms. These forms score surgical performance based on preferred surgical actions and errors observed during surgical procedures^3–5^. Recently, the measurement of psychomotor parameters has become popular. Objective variables, such as hand movement trajectory, speed, consistency, coordination, eye-scanning trajectory, and pupil dilatation^6–12^, can remove the bias from subjective judgement^4^. Besides hand and instrument motions, it is now possible to monitor the surgeon’s eye movements using sophisticated tracking systems^13^^,^^14^. Previous studies^15–17^ demonstrated that distinct hand and eye movement patterns can be observed between experienced and novice surgeons; however, evidence of combined hand and eye movements has not been explored fully. This is not a trivial problem, as eye–hand coordination is the foundation of surgical skills. The goals of the present study were to investigate whether surgical experts have eye–hand coordination different to that of novices.

Early work examining eye–hand coordination outside of the surgical domain was done by Flanagan and Johansson in 2003^15^. They found that, in reaching and grasping tasks, the participants’ eye gaze travelled to the target about 550 ms before their hand movements^15^^,^^18^.

Pre-action eye shift was also found in athletes, particularly basketball players, during free-throw shooting^19^^,^^20^. A successful shooting trial is often preceded by a long gaze over the key hot spots surrounding the basketball net. Vickers^19^ named this long gaze period the quiet eye phase, which could last from 1200 to 1500 ms, whereas in an unsuccessful shooting, the quiet eye phase could be much shorter. In a laparoscopic setting, Wilson and colleagues^21^ also found a similar gaze strategy, with expert surgeons displaying a longer quiet eye phase than novices. Although Wilson used the same terminology as Vickers^18^, Wilson’s definition of quiet eye phase focused more on gaze disengagement from the previous task rather than gaze early engagement of the current task, which was used in Vicker’s basketball free-throw task^19^.

In the present controlled laboratory study, both disengagement and early engagement in a simulated operation were included to investigate which yielded a better outcome in describing eye–hand coordination patterns between expert and novice. The laparoscopic task included three separate phases: reaching and grasping, transporting and loading an object, and bringing the instrument back to the home station. The task was performed by three groups of participants with different levels of surgical expertise^22^.

It was hypothesized that highly experienced surgeons would complete the task more quickly than both novices and intermediate surgeons. With the shorter task time, experts would perform more proactive eye movements (disengage rapidly from the previous subtask and engage early on the current one) and display longer duration of action-related fixation (eye gaze fixation on a target before the instrument reaches the target) than novices, which would further suggest that experts have, over time, acquired a ‘smart’ strategy to maintain their gaze on the key surgical area to guide their hands during task performance.

## Methods

This study was conducted at the Department of Surgery of the University of British Columbia (UBC) and the Medical Imaging Research Laboratory at Simon Fraser University. Ethics approval was obtained from the research ethics board of these two universities before the recruitment of human subjects. Twelve surgeons, including five experienced (more than 10 years of working as faculty surgeons, each having performed over 300 laparoscopic procedures) and seven intermediate surgeons (fellows and residents, each having performed less than 100 laparoscopic procedures) were recruited from UBC. Fourteen university students (with no surgical training) were recruited from Simon Fraser University. All participants had normal or corrected-to-normal vision, and most reported being right-handed (21 : 5 participants). Informed, written consent was obtained from each participant before entering the study.

### Surgical experience score

Each participant was surveyed with regard to their surgical experience in performing up to 12 different laparoscopic procedures, undertaken as a surgeon and as an assistant. Surgical experience was quantified by grouping the number of procedures performed into one of five categories (0–1, 2–5, 6–10, 11–15, more than 15), and by scoring 1, 2, 3, 4 or 5 points for each category (*Appendix S1*). For an expert who performed over 30 of each of 12 laparoscopic procedures, the maximum raw points earned was 120 points (60 points as a surgeon plus 60 points as an assistant). A surgical experience score was calculated for each participant by normalizing the raw points as a percentage, using the equation:

surgical experience score=raw points×100/120.

### Apparatus

**Fig. 1:**
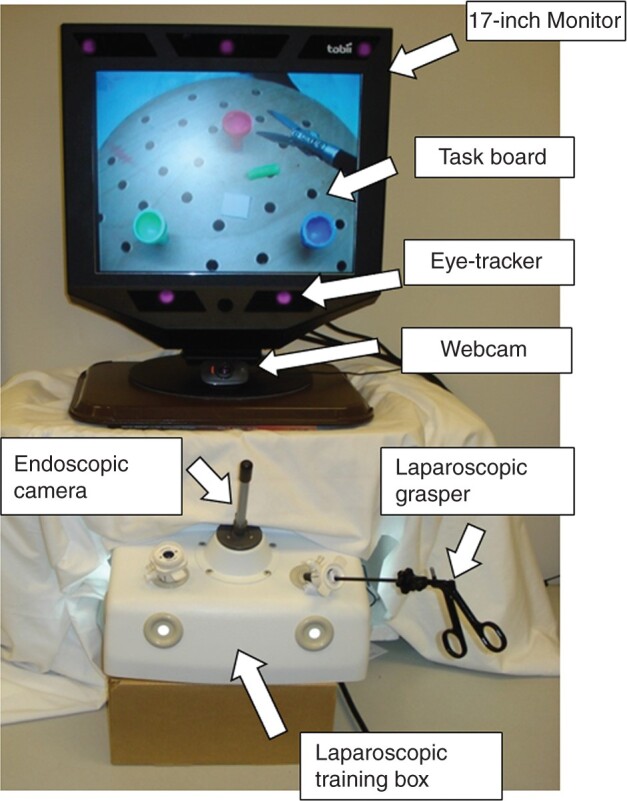
Experimental set-up

The same laparoscopic training and data recording systems were used at both data collection sites. The laparoscopic training box (Laparoscopic Trainer; 3-D Technical Services, Franklin, OH, USA) has two ports for connecting laparoscopic instruments and one endoscopic camera to capture the surgical video (*Fig. 1*).

On the top of the training box, a remote eye-tracker (Tobii 1750; Tobii Technology, Danderyd, Sweden) was placed, about 60–70 cm away from the standing subject. The eye-tracker recorded eye movements on a 17-inch LCD monitor at 50 Hz via three infrared sensors built into the base of the monitor. The Tobii 1750 tracker detects gaze on the monitor with errors less than 1° of the visual field, which is sufficient to distinguish the surgical anatomy displayed on the monitor. A web camera (C525 HD Webcam; Logitech, Fremont, CA, USA) was placed below the eye-tracker to record the surgeon’s face. Video taken by this web camera was used for checking any lost eye-tracking data, such as eye blinks and large head movements.

### Task

**Fig. 2:**
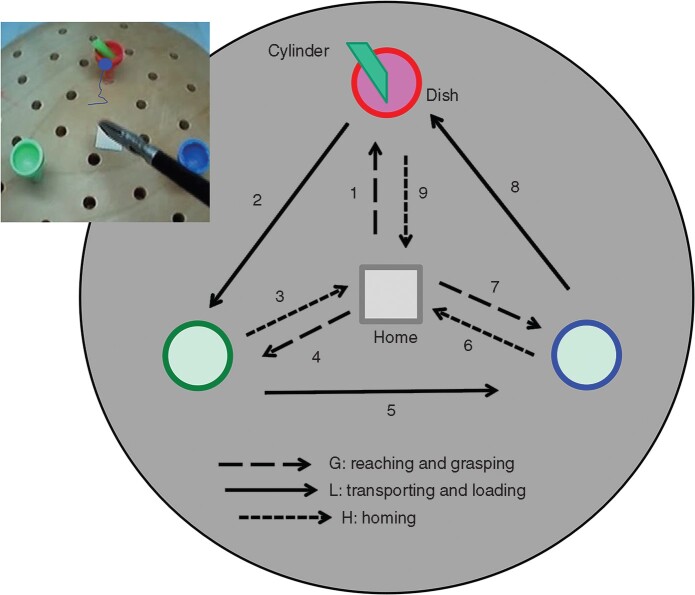
Subtasks and steps of simulation task The subject’s eye gaze moved to the red dish before the instrument, showing an early-engagement event.

A task board was placed inside the training box. Each participant was required to use a laparoscopic grasper (Ethicon Endo-Surgery, Cincinnati, OH, USA) to move an object (a plastic green cylinder, 3 mm wide and 7 mm long) over three dishes (10 mm in diameter) in a predetermined order (*Fig. 2*). One complete trial took 60–90 s. This simple but sequential task was chosen as it comprised basic features of laparoscopic surgery, including reaching, grasping, transporting, and loading; it required precision on the manipulation while controlling the laparoscopic tool under video guidance. Yet, the task included separable steps and actions allowing measurement of action-related eye movements.

Each participant had 5 min to practise the task with their preferred hand to familiarize themselves with both the simulation and the required task. Next, the eye-tracking system calibration process was performed with each participant. Data collection began by asking the participant to perform five trials, with a short break in between each trial. The participant’s eye movements, surgical process, and facial expressions were recorded.

### Data synchronization

Three data streams were synchronized over the time frames. The surgical scene video was captured by a television tuner card (Hauppauge HVR2250; Hauppauge Computer Works, Hauppauge, NY, USA) and displayed on the Tobii monitor using Clearview 2.7.0 (Tobii Technology, Danderyd, Sweden) eye-tracking data analysis software, where eye-tracking signals were integrated and displayed. The surgical scene videos were recorded at a lower resolution (352 × 288 pixels) than that of the Tobii monitor (1240 × 1024 pixels). Methods for aligning videos with different resolutions have been reported elsewhere^8^. The web camera recorded the participant’s face at a speed of 30 frames per s, whereas the Tobii eye-tracking system recorded eye motion data at a speed of 50 frames per s. To establish temporal correspondence among three video streams (surgical video, eye-tracking signals, and facial webcam video), camera flashes were introduced at the start and end of the trial. The short 4-ms flashing lights captured by all cameras were used as markers to adjust the temporal correspondence between videos from different sources.

### Data analyses

After the videos and eye-tracking signals had been synchronized in time and spatial coordinates, the eye-scanning trajectories were overlaid on the surgical scene video (*Fig. 2*). The locations of the instrument tip during these videos were identified by a custom-designed algorithm developed using C++ (Microsoft Visual Studio, Microsoft, Redmond, WA, USA) and OpenCV Library^23^.

The total task time (TTT) was defined as the time between the moment the instrument grasper departed from its home station to the moment of its return to the home station from the red dish (*Fig. 2*). Each trial contained nine steps, and the onset of each step was separated by the moment when the instrument grasper departed from the home station or the dish for holding the cylinder.

**Fig. 3:**
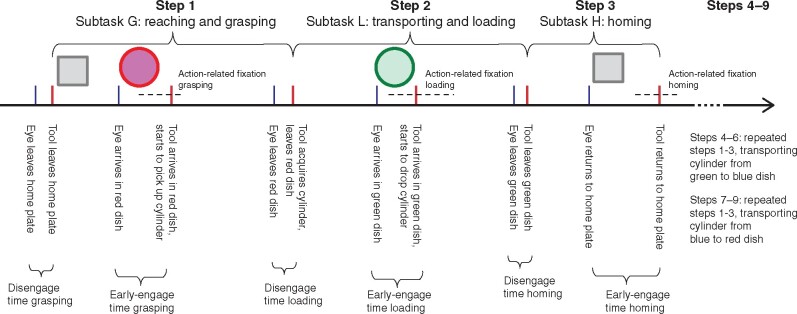
Key events and time variables at each step

The nine trial steps comprised three types of subtask (*Fig. 3*): reaching and grasping the cylinder (G); transporting and loading the cylinder into a dish (L); and bringing the instrument to the home station (homing, H)^24^^,^^25^. The subtask time was calculated by averaging the time used for each type of subtask. The H subtask was less demanding (low in task difficulty) than the G and L subtasks.

For each step, the following events were annotated on the eye-tracking video for each participant (*Table 1*). The gaze of a participant might begin to disengage from the home station before the surgical instrument actually leaves the home station. The disengage time (DET) from a previous subtask was calculated by subtracting event 2—event 1; the DET was similar to the proactive gaze movement in Flanagan and Johansson’s study^15^. Moreover, the surgeon’s gaze may arrive at the target before the instrument during the current subtask. When this occurred, an early-engage time (EET) was recorded, which was calculated by subtracting event 4—event 3. During event 4, when the instrument grasper was either in the act of grasping, releasing the object (cylinder), or touching the home station, the duration of the final fixation associated with this action was further examined (gaze within 1° of the visual angle for a minimum of 120 ms). This measurement was referred to an the action-related fixation (ARF) on the current action in hand, which is related to the quiet eye measure defined by Vickers^19^, and is also related to the target locking measure used by Wilson and colleagues^26^ in analysing sequential tasks such as surgery. An illustration has been created to describe DET, EET, and ARF (*Fig. 3*).

**Table 1:** Video analysis events and statistical measures

	Explanation/calculation
**Events**	
1	Gaze of subject leaves home station or dish
2	Grasper (tool) leaves home plate or dish
3	Gaze of subject arrives at home plate or dish
4	Grasper (tool) arrives at home station or dish; at this moment, the grasper starts to grasp (subtask G), release the cylinder (subtask L), or to touch down on the home station (subtask H)
**Measures**	
Total task time	Time between grasper leaving home station for the first time and it returning to home station on the third time
Subtask time grasping	Time between grasper leaving home station and it leaving dish with cylinder
Subtask time grasping	Time between grasper arriving at next dish and it leaving dish after releasing cylinder
Subtask time loading	Time between grasper leaving second dish and it resting on home station
Disengage time	Event 2 – event 1
Early-engage time	Event 4 – event 3
Action-related fixation	Fixation at event 4

### Statistical analysis

Results are reported as mean(s.d.) unless stated otherwise. Correlation analyses between surgical experience score, DET, EET, and ARF were performed using the Pearson formula in SPSS^®^ version 22.0 (IBM, Armonk, NY, USA). The dependent variables of task time, DET, EET, and ARF were subjected to a three-group (expert, intermediate, novice) × three subtask (G *versus* L *versus* H) mixed ANOVA with repeated measures on the subtask. *P* ˂ 0.050 was considered significant.

## Results

Demographics of subjects are shown in *Table 2*. Twenty-six participants performed a total of 130 trials (5 trials for each subject). However, eye-tracking data were recorded inappropriately in 25 trials (3 experts, 3 intermediates, 19 novices) owing to large head movements while performing the task or invalid data for tracking fixation (less than 70 per cent of the TTT). Two novice participants’ data (10 trials) were removed completely. Of the 105 valid trials performed by 24 participants (5 experts, 7 intermediates, 12 novices), there should be a total of 945 steps (105 trials with 9 steps each). However, there were five invalid steps where the gaze signal was missing and eye–hand coordination variables could not be obtained because the calculation required both operating and observing signals to be valid simultaneously. Therefore, data from 940 valid steps were entered into the analysis.

**Table 2:** Demographic information for three groups of participants

	Expert	Intermediate	Novice
	(*n =* 5)	(*n =* 7)	(*n =* 12)
Current position	Faculty surgeons	Fellows and residents	Novices
Mean age (years)	49.6	31.0	24.2
Sex ratio (M : F)	5 : 0	5 : 2	8 : 4
Years of experience in surgery	23	4	0
No. of laparoscopic procedures	> 150	30–60	< 1
Mean(s.d.) surgical experience score	81.5(13.7)	43.2(12.8)	20.8(1.2)

### Task time

Testing on the TTT revealed a significant difference over three subject groups (*F*_2,21_ = 10.488; *P* = 0.001) (*Table 3*). Specifically, expert surgeons performed the task in a shorter time (mean(s.d.) 21.3(4.8) s) than the intermediate (36.3(6.9) s) and the novice (40.3(9.1) s) groups. A *post*  *hoc* (Bonferroni correction) test revealed that significant differences were present between the expert and intermediate (*P* = 0.011), and expert and novice (*P* = 0.001), groups, but not between the intermediate and novice groups (*P* = 0.862).

**Table 3:** ANOVA results for task time and eye-tracking variables during various subtasks among expert, intermediate, and novice surgeons

	Expert	Intermediate	Novice	Group		Subtask		Interaction
	(*n =* 5)	(*n =* 7)	(*n =* 12)	*P*	*η_p_^2^*	*P*	*η_p_^2^*	*P*
**Task time (s)**								
Reaching and grasping	2.017(0.619)	3.637(1.347)	3.781(1.035)	0.001	0.49	<0.001	0.584	<0.001
Transporting and loading	2.960(0.752)	4.579(0.771)	6.296(1.853)					
Homing	2.069(0.321)	3.878(0.551)	3.251(0.483)					
**Disengage time (ms)**								
Reaching and grasping	44(120)	–173(157)	–27(104)	0.003	0.43	<0.001	0.439	<0.001
Transporting and loading	–26(96)	–196(145)	–413(218)					
Homing	–20(63)	–199(179)	–450(215)					
**Early-engage time (ms)**								
Reaching and grasping	1316(273)	2052(967)	2641(1076)	0.007	0.37	<0.001	0.452	0.259
Transporting and loading	1194(364)	2186(697)	2483(844)					
Homing	934(649)	1190(316)	1345(486)					
**Action-related fixation (ms)**								
Reaching and grasping	1003(108)	1113(289)	1037(225)	0.367	0.09	0.001	0.304	0.158
Transporting and loading	1244(182)	1247(304)	1412(327)					
Homing	889(120)	1155(226)	852(311)					
**Action-related fixation/subtask time (%)**								
Reaching and grasping	55(21)	34(15)	30(12)	0.001	0.47	0.032	0.171	0.765
Transporting and loading	45(14)	28(9)	25(10)					
Homing	43(5)	30(6)	26(9)					

Values are mean(s.d.).

**Fig. 4:**
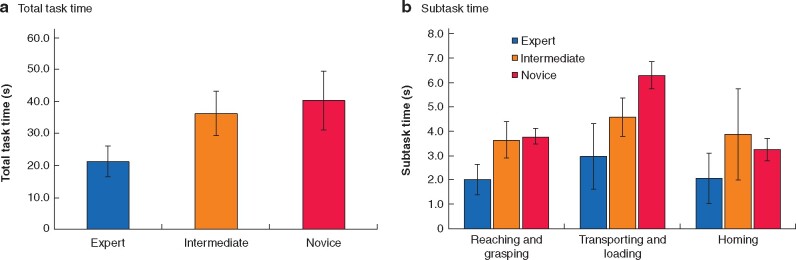
Total task and subtask times for expert, intermediate, and novice surgeons **a** Total task time and **b** subtask time. Values are mean(s.d.).

Mixed ANOVA of the subtask time revealed significant differences among the groups (*F*_2,21_ = 9.914; *P* = 0.001), subtasks (*F*_2,42_ = 29.502; *P* < 0.001), and the interaction between subtask and group (*F*_2,42_ = 7.481; *P* < 0.001). Experts spent more time performing the transporting and loading subtasks than the reaching and grasping subtasks and bringing the grasper to the home station (*Fig. 4*). These patterns were more prominent in the intermediate and novice surgeons.

### Disengage time and early-engage time

**Fig. 5:**
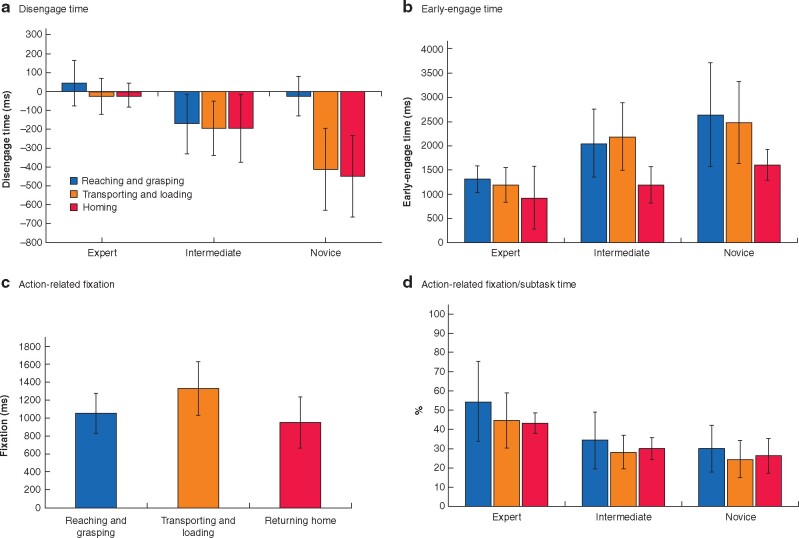
Comparison of task times for expert, intermediate, and novice surgeons over three subtasks **a** Disengage time and **b** early-engage time by subtask and surgeon experience, **c** action-related fixation over three subtasks, and **d** action-related fixation as a percentage of subtask time for expert, intermediate, and novice surgeons. Values are mean(s.d.).

Mixed ANOVA of the DET revealed significant differences among the groups (*F*_2,21_ = 6.268; *P* = 0.007), subtasks (*F*_2,42_ = 16.400; *P* < 0.001), and the interaction between subtask and group (*F*_2,42_ = 11.367; *P* < 0.001). Experts managed to disengage their eyes from the previous subtask 44 ms before the instrument started to move during the reaching and grasping subtask (*Fig. 5a*). Gaze disengagement occurred 26 ms after the actual surgical instrument movement, whereas transporting and loading, and gaze disengagement occurred 20 ms after bringing the instrument back to the home station. Averaged over three subtasks, the experts’ eye was moving simultaneously with the tool (–1(93) ms). Intermediate surgeons (–189(160) ms) and novices (–296(179) ms) were not able to disengage their gaze from the previous subtask before the instrument was moved towards the next target; generally, novices’ gaze shifts were more delayed after surgical tool movement, especially during the subtask of bringing the instrument back to the home station.

The result of statistical analysis of eye EET on the current subtask was different from that of DET. Mixed ANOVA of the EET revealed a significant difference among the groups (*F*_2,21_ = 6.268; *P* = 0.007) and subtasks (*F*_2,42_ = 17.336; *P* < 0.001) but not on their interaction (*F*_2,42_ = 1.399; *P* = 0.259). All participants managed to move their eyes to the current target more than 1000 ms before the tool was actually moved (*Fig. 5b*). In novices, the mean EET was 2243(745) ms, significantly longer than that for intermediate (1809(589) ms) and expert (1148(429) ms) surgeons. All participants were similar in that their eyes displayed early engagement with the target as the subtasks moved from reaching and grasping to transporting and loading, then to returning the grasper to the home station (*Fig. 5b*).

### Action-related fixation

ANOVA for the duration of ARF revealed significant differences for the subtasks (*F*_2,42_ = 9.151; *P* = 0.001) but not for the groups (*F*_2,21_ = 1.052; *P* = 0.369) or the interactions between them (*F*_2,42_ = 1.748; *P* = 0.158). Participants had significantly longer fixation times on the transporting and loading subtask (1329(297) ms) compared with reaching and grasping (1052(223) ms) and returning home (948(284) ms) subtasks (*Fig. 5c*). Expert (1045(201) ms), intermediate (1172(267) ms), and novice (1100(369) ms) surgeons all fixated on the target associated with the upcoming tasks.

The ratio of ARF to duration of subtask was calculated. There was a significant difference among the groups (*F*_2,21_ = 9.484; *P* = 0.001) and subtasks (*F*_2,42_ = 4.333; *P* = 0.032), but not their interactions (*F*_2,42_ = 0.377; *P* = 0.765). Experts fixated on the target for a greater portion of the time compared with intermediates and novices (*Fig. 5d*); this phenomenon was more prominent in the reaching and grasping tasks than when returning the tool to the home position.

### Correlations between surgical experience score and eye matrix

**Fig. 6:**
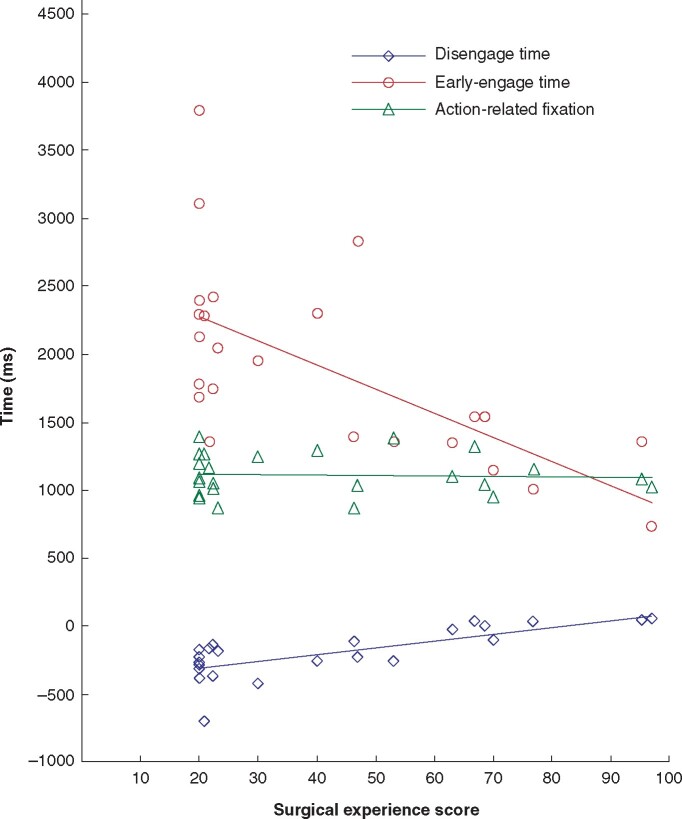
Spatial plots showing correlation between surgical experience scores and disengagement time, early-engagement time, and action-related fixation Linear correlations: disengagement time, *r* = 0.743; early-engagement time, *r* = – 0.649; and action-related fixation, *r* = – 0.067.

Correlation coefficients and significant test outputs are presented in *Fig. 6*. The correlation between surgical experience score and DET, EET, and ARF is visualized in a scatter plot. Specifically, a strong, significant, and positive correlation was noted between surgical experience score and the DET (*r* = 0.743; *P* < 0.001), and a strong, significant, and negative correlation between surgical experience score and the EET (*r* = –0.649; *P* = 0.001); however, there was no significant correlation between surgical experience score and the ARF (*r* = –0.067; *P* = 0.755).

## Discussion

The focus of this study was to determine whether expert surgeons have developed some unique spatiotemporal characteristics in their eye–hand coordination that build the foundation of their superior performance. Specifically, three variables were examined intentionally to describe eye–hand coordination features: eye disengagement from a previous subtask, eye early engagement to an ongoing (current) subtask, and the fixation associated with an ongoing action with instrument in hand, as suggested previously^15^^,^^22^^,^^26^.

Findings from eye disengagement analyses indicated that expert surgeons were able to shift their gaze away from the previous subtask about 300 and 200 ms earlier than the novices and intermediate surgeons respectively. From this, it is deduced that expert surgeons were confident in their performance of the previous subtask; as a result, they could shift their attention by disengaging their eyes from the previous trget to the current target while simultaneously moving their hands. However, novices and surgeons in training are not as confident in their performance of the previous subtask and need extra time to double-check their work, even when the surgical instrument is already moving to the next target.

A longer DET was recorded for more demanding previous subtasks. When the previous subtask was relatively easy, such as homing, the surgeons disengaged and shifted their gaze to the next target with only a short delay. If the previous subtasks were challenging, such as grasping or loading, all surgeons seemed to need to focus longer on the previous subtask even after the current subtask had been initiated. Results suggested that the surgeons’ visual attention was regulated by the level of task requirement of the previous subtask. Thus, if the level of task requirement remained the same between tasks, the ability to disengage eyes from the previous action may be a promising behavioural marker for describing surgical expertise.

Early engagement of eyes to the current subtask was observed in all operators. This can be explained by the longer task time taken by novices. With novices, the slower the tool transportation time during a particular subtask, the longer the EET recorded. After their first eye gaze on the target, the novice often looked back at the tool during tool transportation. Human operators (even novices) can perform rapid eye scanning among various visual sites with a saccade speed up to 900° per s^27^, much faster than hand movement. With a longer transportation time, novices checked the target and the instrument tip many times rather than fixating on the target. In contrast, expert surgeons transported their tools considerably faster than novices. Yet, the experts managed to scan over the target promptly before gazing at the tool. Once experts fixated on the target, they seldom moved back to the tool. This phenomenon is consistent with Law’s finding^28^ that novices’ eyes were spending longer on the surgical instrument tips rather than on the target. It was noted that the standard deviation of EETs was smaller in experts (429 ms) than in novice (745 ms) and intermediate (589 ms) surgeons. This means that experts gazed on the target before their hand with a high consistency over trials, whereas novices had a lower degree of consistency in performing the early target scan, partially owing to their frequent movement back and forth between the target and the tool. Therefore, merely calculating the EET may be not sufficient to reflect surgical expertise.

There was no significant difference in the ARF duration between different surgical groups, which differs from findings of other studies^19^^,^^26^ analysing the quiet eye phase. All operators in the present study, including surgeons and novices, fixated on the target for approximately the same length of time before grasping, loading the cylinder, or touching the home station.

Considering the results from the correlation analysis, it is concluded that eye disengagement from the previous subtask is a better behavioural indicator for surgical expertise when evaluating a compound surgical task comprising a series of sequential subtasks. Further analyses would be needed when choosing the EET or ARF duration as indicators of expertise.

This study has some limitations. First, the simulation tasks used in the study are far from being a perfect representation of real-life surgical procedures. The laparoscopic task, with clean landmarks to separate steps and subtasks, was designed to facilitate data analysis on the eye–hand coordination of the surgeons. Caution will be needed when applying the findings to a real surgical context. Second, the novices included in this study were university students who did not receive any medical training. Their behaviours may not be equivalent to those of medical trainees. In the future, the authors would like to continue to gather more eye-tracking data from real surgical scenarios, which could ultimately be used to further investigate the relationship between eye–hand coordination and surgical expertise.

## Supplementary Material

zrab068_Supplementary_Data
