# Supplementary material for: Action-related eye measures to assess surgical expertise
Source: BJS Open. 2021 Sep 3;5(5):zrab068. doi: 10.1093/bjsopen/zrab068 (PMC8413367; doi:10.1093/bjsopen/zrab068)
Supplement: zrab068_Supplementary_Data [file zrab068_supplementary_data.doc]

**Surgical Experience Questionnaire**

Eye-tracking Project

**No: _________ Date: __________________________________**

|  |  | | | | |  | | | | |  | | | | | | | |  | | | |  | | | |  | | | |  | | | | |
| --- | --- | --- | --- | --- | --- | --- | --- | --- | --- | --- | --- | --- | --- | --- | --- | --- | --- | --- | --- | --- | --- | --- | --- | --- | --- | --- | --- | --- | --- | --- | --- | --- | --- | --- | --- |
| **Gender**: | Male | | | Female | | |  | | | | | **Age:** | | | |  | | | |  | | | **Handedness:** | | | | | | R | L | | |  | | |
|  | |  | | | |  | | | | |  | | | | | | | |  | | | |  | | | |  | | | |  | | | | |
| **Level of Training:** | | | | | Student | | | | R1 | | | | R2 | | | | | | R3 | | | R4 | | | R5 | | | Fellow | | | | Faculty | | | |
|  | |  | | | |  | | | | |  | | | | | | | |  | | | |  | | | |  | | | |  | | | | |
| **Number of Years doing Laparoscopic Surgery:** 0-1 2-3 4-6 7-9 10+ | | | | | | | | | | | | | | | | | | | | | | | | | | | | | | | | | | | |
|  | |  | | | |  | | | | |  | | | | | | | |  | | | |  | | | |  | | | |  | | | | |
| **Please circle the number of times you have performed each of the following procedures as a** | | | | | | | | | | | | | | | | | | | | | | | | | | | | | | | | | | | |
| **Surgeon** *(in column 1)* **and as an assistant** *(in column 2).* | | | | | | | | | | | | | | | | | | | | | | | | | | | | | | | | | | | |
|  | | |  | | |  | | | |  | | | | | | | | |  | | | |  | | | |  | | | |  | | | | |
|  | | |  | | | | | | | **Performed as Surgeon** | | | | | | | | | | | | | |  | | **Performed as Assistant** | | | | | | | | | |
| **Procedure** | | | | | | | |  | | **Frequency** | | | | | | | | | | | | | |  | | **Frequency** | | | | | | | | | |
|  | | | | | | | |  | |  | | | | |  | |  | | | |  | | |  | |  | | |  | |  | | |  | |
| 1) Laparoscopic Cholecystectomy: | | | | | | | | 0-1 | | 2-5 | | | | 6-10 | | | | 11-15 | | | >15 | | | 0-1 | | 2-5 | | | 6-10 | | 11-15 | | | >15 | |
|  | | | | | | | |  | |  | | | |  | | | |  | | |  | | |  | |  | | |  | |  | | |  |  |
| 2) Diagnostic Laparoscopy: | | | | | | | | 0-1 | | 2-5 | | | | 6-10 | | | | 11-15 | | | >15 | | | 0-1 | | 2-5 | | | 6-10 | | 11-15 | | | >15 |  |
|  | | | | | | | |  | |  | | | |  | | | |  | | |  | | |  | |  | | |  | |  | | |  |  |
| 3) Laparoscopic Appendectomy: | | | | | | | | 0-1 | | 2-5 | | | | 6-10 | | | | 11-15 | | | >15 | | | 0-1 | | 2-5 | | | 6-10 | | 11-15 | | | >15 |  |
|  | | | | | | | |  | |  | | | |  | | | |  | | |  | | |  | |  | | |  | |  | | |  |  |
| 4) Lap Nissen Fundoplication: | | | | | | | | 0-1 | | 2-5 | | | | 6-10 | | | | 11-15 | | | >15 | | | 0-1 | | 2-5 | | | 6-10 | | 11-15 | | | >15 |  |
|  | | | | | | | |  | |  | | | |  | | | |  | | |  | | |  | |  | | |  | |  | | |  |  |
| 5) Laparoscopic Splenectomy: | | | | | | | | 0-1 | | 2-5 | | | | 6-10 | | | | 11-15 | | | >15 | | | 0-1 | | 2-5 | | | 6-10 | | 11-15 | | | >15 |  |
|  | | | | | | | |  | |  | | | |  | | | |  | | |  | | |  | |  | | |  | |  | | |  |  |
| 6) Laparoscopic Bowl Resection: | | | | | | | | 0-1 | | 2-5 | | | | 6-10 | | | | 11-15 | | | >15 | | | 0-1 | | 2-5 | | | 6-10 | | 11-15 | | | >15 |  |
|  | | | | | | | |  | |  | | | |  | | | |  | | |  | | |  | |  | | |  | |  | | |  |  |
| 7) Laparoscopic Adrenalectomy: | | | | | | | | 0-1 | | 2-5 | | | | 6-10 | | | | 11-15 | | | >15 | | | 0-1 | | 2-5 | | | 6-10 | | 11-15 | | | >15 |  |
|  | | | | | | | |  | |  | | | |  | | | |  | | |  | | |  | |  | | |  | |  | | |  | |
| 8) Laparoscopic Nephrectomy: | | | | | | | | 0-1 | | 2-5 | | | | 6-10 | | | | 11-15 | | | >15 | | | 0-1 | | 2-5 | | | 6-10 | | 11-15 | | | >15 | |
|  | | | | | | | |  | |  | | | |  | | | |  | | |  | | |  | |  | | |  | |  | | |  | |
| 9) Laparoscopic Bariatric surgery: | | | | | | | | 0-1 | | 2-5 | | | | 6-10 | | | | 11-15 | | | >15 | | | 0-1 | | 2-5 | | | 6-10 | | 11-15 | | | >15 | |
|  | | | | | | | |  | |  | | | |  | | | |  | | |  | | |  | |  | | |  | |  | | |  | |
| 10) Lap Inguinal Hernia Repair: | | | | | | | | 0-1 | | 2-5 | | | | 6-10 | | | | 11-15 | | | >15 | | | 0-1 | | 2-5 | | | 6-10 | | 11-15 | | | >15 | |
|  | | | | | | | |  | |  | | | |  | | | |  | | |  | | |  | |  | | |  | |  | | |  | |
| 11) Lap Incisional Hernia Repair: | | | | | | | | 0-1 | | 2-5 | | | | 6-10 | | | | 11-15 | | | >15 | | | 0-1 | | 2-5 | | | 6-10 | | 11-15 | | | >15 | |
|  | | | | | | | |  | |  | | | |  | | | |  | | |  | | |  | |  | | |  | |  | | |  | |
| 12) Others: | | | | | | | | 0-1 | | 2-5 | | | | 6-10 | | | | 11-15 | | | >15 | | | 0-1 | | 2-5 | | | 6-10 | | 11-15 | | | >15 | |
|  | | | | | | | |  | | | | | | | | | | | | | | | | | | | | | | | | | | | |

Your contact email: (optional)_______________________________________
